# Inhibition of ROS1 activity with lorlatinib reversibly suppresses fertility in male mice

**DOI:** 10.1111/andr.13808

**Published:** 2024-11-20

**Authors:** Yuki Oyama, Kentaro Shimada, Haruhiko Miyata, Rie Iida‐Norita, Chihiro Emori, Maki Kamoshita, Seiya Oura, Ryohei Katayama, Martin M. Matzuk, Masahito Ikawa

**Affiliations:** ^1^ Graduate School of Pharmaceutical Sciences Osaka University Suita Osaka Japan; ^2^ Department of Experimental Genome Research, Research Institute for Microbial Diseases Osaka University Suita Osaka Japan; ^3^ Division of Experimental Chemotherapy, Cancer Chemotherapy Center Japanese Foundation for Cancer Research Koto‐ku Tokyo Japan; ^4^ Center for Drug Discovery Baylor College of Medicine Houston Texas USA; ^5^ Department of Pathology & Immunology Baylor College of Medicine Houston Texas USA; ^6^ The Institute of Medical Science The University of Tokyo Minato‐ku Tokyo Japan; ^7^ Center for Infectious Disease Education and Research Osaka University Suita Osaka Japan

**Keywords:** fertilization, male contraceptive, small molecule inhibitors, sperm maturation

## Abstract

**Background:**

Inhibition of sperm maturation in the epididymis is a promising post‐testicular strategy for short‐acting male contraceptives. It has been shown that ROS1, a receptor tyrosine kinase expressed in the epididymis, is essential for epididymal differentiation, sperm maturation, and male fertility in mice. However, it is unknown if inhibition of ROS1 suppresses male fertility reversibly.

**Objectives:**

Our study aimed to investigate the effects of ROS1 inhibitor administration in male mice on sperm function and fertility.

**Materials and methods:**

We used lorlatinib, an anti‐cancer drug that inhibits ROS1. We treated 10‐week‐old sexually mature male mice with lorlatinib for 3 weeks and performed fertility tests, histological staining, in vitro fertilization, sperm motility analyses, and immunoblot analyses. We also performed the same analyses 3 weeks after discontinuing the lorlatinib treatment.

**Results:**

Inhibition of ROS1 for 3 weeks suppressed male fertility. Lorlatinib‐treated mice showed no overt abnormalities in testicular sections, but epithelium maintenance of the epididymal initial segment was impaired. Accordingly, the levels of OVCH2, RNASE10, and ADAM28, which are expressed in the epididymis, decreased. Spermatozoa from the lorlatinib‐treated mice lost their ability to bind to the zona pellucida, and ADAM3 processing was abnormal. Sperm motility was also impaired in the lorlatinib‐treated mice. These impairments were recovered 3 weeks after discontinuing the drug treatment.

**Discussion and conclusion:**

Inhibition of ROS1 with lorlatinib suppressed sperm maturation and male fertility reversibly. Future exploration of molecules that specifically target ROS1 and the ROS1 pathway in the epididymis may lead to the development of safe and reversible male contraceptives.

## 1 INTRODUCTION

Global statistics show that about 50% of pregnancies are unwanted, and more than half of these result in abortion.^1^ To improve this situation, the development and dissemination of effective contraceptive methods is urgently needed. While there are more than 10 different methods of birth control for women, including contraceptive pills and intrauterine devices, men have had limited options: condoms and vasectomies. Dependence on women for contraception is undesirable from the perspective of gender equality, and the development of male contraceptives that allow men to control their own fertility is desired.

JQ1, a molecule that targets BRDT (bromodomain, testis‐specific), has the potential to be a reversible male contraceptive.^2^ However, because JQ1 targets spermatogenesis which takes approximately 5 weeks, it takes longer time for JQ1 to show contraceptive effect, and JQ1 also targets other BRDs, BRD2, BRD3, and BRD4. Similarly, prolonged inhibition of serine–threonine kinase 33 (STK33) kinase activity in testicular spermatozoa causes reversible contraceptive effects.^3^ In contrast to targeting spermatogenesis, there are several trials that target sperm motility such as soluble adenylyl cyclase^4^ and sperm maturation such as sperm‐specific calcium‐dependent phosphatase (calcineurin) for short‐acting contraception.^5^ For example, calcineurin inhibitors, cyclosporin A (CsA) and FK506, render male mice infertile after 2 weeks of treatment due to impaired sperm maturation in the epididymis, suggesting that specific inhibition of sperm maturation may lead to the development of male contraceptives that function quickly and effectively.

*Ros1* (Ros1 proto‐oncogene) is a proto‐oncogene, which encodes a receptor tyrosine kinase whose aberrant fusion protein is known to cause lung cancer.^6^ ROS1 is expressed in the initial segment (IS) of the epididymis in mice, and *Ros1* knockout (KO) male mice are infertile due to abnormal epithelium differentiation of the IS and impaired sperm maturation.^7^ The ligand for ROS1 was unknown for many years, but recently the complex of NELL2 (NEL‐like 2) and NICOL1 (NELL2 interacting cell ontogeny regulator 1) was identified as the ROS1 ligand.^8^, ^9^ NELL2/NICOL1 is derived from testicular germ cells and acts as a lumicrine factor because it migrates through the lumen from the testis to the epididymis to promote IS differentiation. Blocking the supply of the lumicrine factor by ligating the duct between the testis and epididymis (efferent duct) causes regression of once differentiated IS in adult rats and mice,^10^, ^11^ suggesting that NELL2/NICOL1 can be a target of male contraceptives. Alternatively, targeting the kinase activity of ROS1 can be a strategy for inducing contraception. Crizotinib is an anti‐cancer drug that inhibits both ROS1 and ALK (anaplastic lymphoma kinase). In contrast to *Ros1* expression in the epididymis, no expression of *Alk* has been detected in mouse epididymis by quantitative reverse transcription‐polymerase chain reaction (qRT‐PCR).^12^ In a report that analyzed the effects of crizotinib on mouse epididymis, treatment of male mice with crizotinib for 12 days did not show IS regression or decreased fertility,^12^ making it unclear whether the ROS1 pathway can be a target for male contraceptives.

In our study, we analyzed the effects of lorlatinib, a different anti‐cancer drug targeting ROS1 and ALK, to determine whether ROS1 pathway can be an effective target for male contraceptives. Our results show that ROS inhibition suppresses sperm maturation and male fertility reversibly in mice.

## 2 MATERIALS AND METHODS

### 2.1 Animals

All animal experiments performed in this study were approved by the Institutional Animal Care and Use Committees of Osaka University in compliance with the guidelines and regulations for animal experiments (#Biken‐AP‐H30‐01 and #Biken‐AP‐R03‐01). Animals were housed in a temperature‐controlled environment with 12 h light cycled and free access to food and water. All mice were purchased from CLEA Japan, Inc. or Japan SLC, Inc.

### 2.2 Drug treatment

Crizotinib and lorlatinib (Shanghai Biochempartner) were diluted in 0.02 N HCl and administrated by oral gavage. C57BL6/N male mice were treated with crizotinib (100 mg/kg oral gavage daily), lorlatinib (10 mg/kg oral gavage daily), or vehicle for 3 weeks. Some mice were euthanized for analyses. The remaining mice in which we discontinued drug administration for 3 weeks were analyzed for recovery.

### 2.3 In vivo fertility test

Before mating sham or drug‐treated male mice with B6D2F1 female mice, female mice were superovulated by injection of pregnant mare serum gonadotropin (PMSG; 5 units, ASKA Pharmaceutical) into the abdominal cavity, followed by injection of human chorionic gonadotropin (hCG; 5 units, ASKA Pharmaceutical) with 48 h intervals. Female mice cohabitated with sham‐ or drug‐treated C57BL6/N males after hCG injection. Plugs were checked 15 h after hCG injection. Eggs were then collected 6 h after checking plugs and treated with 0.33 mg/mL hyaluronidase (Sigma‐Aldrich) for 10 min to remove cumulus cells for observing two pronuclei (2PN).

### 2.4 Histological analysis of caput epididymis

Caput epididymis was fixed with 4% paraformaldehyde in phosphate‐buffered saline (PBS) overnight, immersed in paraffin, and sectioned by a Microm HM325 microtome (Microm) at 5 µm thick. Sections were rehydrated and treated with Mayer's hematoxylin solution (FUJIFILM Wako) for 3 min, followed by treatment with eosin solution for 3 min. Sections were observed using a BX53 microscope (Olympus).

### 2.5 Histological analysis of testis

Testes were fixed in Bouin's solution (Polysciences), embedded in paraffin, sectioned at a thickness of 5 µm on a Microm HM325 microtome (Microm), rehydrated, and treated with 1% periodic acid for 5 min, followed by treatment with Schiff's reagent (FUJIFILM Wako) for 10 min. The sections were then stained with Mayer's hematoxylin solution (FUJIFILM Wako) for 1 min and observed using a BX53 microscope with a DP74 color camera (Olympus).

### 2.6 Analyses of sperm morphology and motility

Spermatozoa from cauda epididymis were suspended in Toyoda, Yokoyama, Hoshi (TYH) capacitating medium (119.37 mM NaCl, 4.78 mM KCl, 1.71 mM CaCl_2_·2H_2_O, 1.19 mM KH_2_PO_4_, 1.19 mM MgSO_4_·7H_2_O, 25.07 mM NaHCO_3_, 1.0 mM Na pyruvate, 5.56 mM glucose, 4 g/L bovine serum albumin [BSA], 0.050 g/L streptomycin, 50,000 units/L penicillin).^13^ For morphology assessment, spermatozoa were placed on Matsunami adhesive slide (MAS)‐coated glass slides (Matsunami Glass) and observed using an Olympus BX53 microscope. Sperm motility was analyzed as described previously.^14^, ^15^ Percentages of motile spermatozoa, average path velocity (VAP), straight‐line velocity (VSL), and curvilinear velocity (VCL) were measured as sperm motility parameters. Spermatozoa were incubated at 37°C for either 10 min (non‐capacitated) or 120 min (capacitated) and placed in glass chambers (Leja). Sperm motility was analyzed using the CEROS II sperm analysis system (software version 1.5; Hamilton Thorne Biosciences).

### 2.7 Immunoblot analysis

Protein lysates of testes, caput, or cauda epididymis were prepared with lysis buffer (20 mM Tris–HCl pH7.5, 50 mM NaCl, 1% TritonX‐100) containing protease inhibitor cocktail (Nacalai Tesque) and phosphatase inhibitor cocktail (Nakalai Tesque). Proteins were separated by SDS‐PAGE under reducing conditions and transferred onto poly (vinylidene fluoride) (PVDF) membranes. After blocking with 5% non‐fat dry milk, membranes were incubated with primary antibodies at the indicated dilution (Table S1) overnight. After washes with Tris‐buffered saline (TBS) containing 0.1% Tween 20 (TBST), membranes were incubated with horseradish‐peroxidase conjugated secondary antibodies for 1 h at room temperature. After washes with TBST, signals were detected using Chemi‐Lumi One Super detection kit (Nacalai Tesque) and Amersham ImageQuant 800 (Cytiva).

### 2.8 Sperm‐ZP binding assay

Sperm‐zona pellucida (ZP) binding assay was performed as previously described.^16^ Oocytes were collected from B6D2F1 female mice and treated with 0.33 mg/mL hyaluronidase (Sigma‐Aldrich) for 10 min to remove cumulus cells. Cumulus‐free oocytes were placed in a 100 µL drop of TYH medium. An aliquot of capacitated spermatozoa (4 × 10^5^ spermatozoa/mL) from sham‐ or drug‐treated male mice which were pre‐incubated in TYH for 120 min were inseminated and incubated for 30 min at 37°C under 5% CO_2_ condition. Eggs were fixed with 0.2% glutaraldehyde and observed with an Olympus IX70 microscope.

### 2.9 In vitro fertilization

In vitro fertilization (IVF) was performed as described previously.^17^ Cauda epididymal spermatozoa were dispersed in a 100 µL drop of TYH medium covered with paraffin oil for 2 h at 37°C under 5% CO_2_ for capacitation. Oocytes were obtained from the oviducts of superovulated females and placed in a 100 µL TYH drop. Cumulus‐free oocytes were prepared the same way as for the sperm‐ZP binding assay. To remove the ZP (ZP‐free), oocytes were treated with 1 mg/mL of collagenase (Sigma‐Aldrich) for 5 min. The capacitated spermatozoa were added to the drops containing cumulus‐intact or cumulus‐free oocytes at a final concentration of 2 × 10^5^ spermatozoa/mL. For the drops containing ZP‐free oocytes, spermatozoa were added at a concentration of 2 × 10^4^ spermatozoa/mL. After 8 h of insemination, the formation of 2PN was observed and counted with an Olympus IX70 microscope.

### 2.10 Statistical analyses

Statistical difference was determined using unpaired *t*‐test by Microsoft Office Excel (Microsoft Corporation). Differences were considered statistically significant if the *p* values were less than 0.05. Data represent the mean ± standard deviation (SD).

## 3 RESULTS

### 3.1 Fertility of sexually mature male mice treated with ROS1 inhibitors

**FIGURE 1:**
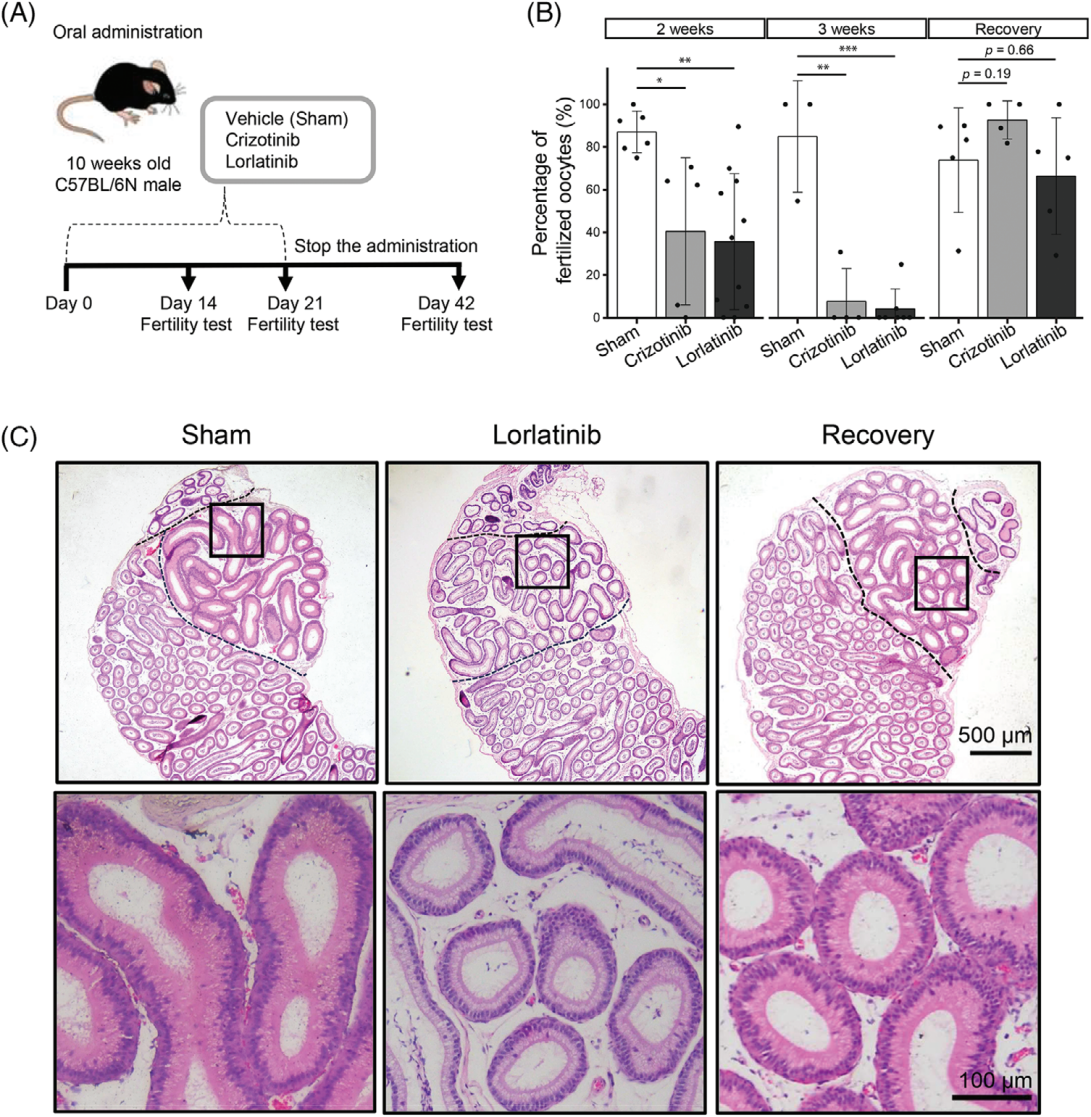
Mice treated with ROS1 inhibitors show reversible fertility loss. (A) Schematic overview of administration experiment. (B) Results of the fertility test. Fertility was examined after 2 and 3 weeks of treatment and 3 weeks after discontinuing treatment (recovery). Data are presented as mean ± SD. Each dot indicates individual mouse. (C) Histological analysis of caput epididymis. *p < 0.05, **p < 0.01, ***p < 0.001 (unpaired *t*‐test).

A ROS1 inhibitor, lorlatinib, was administered orally to 10‐week‐old male mice every day for 3 weeks (Figure 1A). As a comparison, we also administered crizotinib, a different ROS1 inhibitor. No change was observed in mouse body weight during administration (Figure S1A). In vivo fertility tests showed a decrease in male fertility after 2 weeks of lorlatinib and crizotinib treatments, and a more remarkable reduction after 3 weeks (Figure 1B). Specifically, after 3 weeks of lorlatinib treatment, five of seven mice became infertile, while the remaining two mice had reduced fertility (one mouse had a 4.2% fertilization rate and the other had a 25.0% rate). Subsequently, fertility of the male mice treated with either lorlatinib or crizotinib for 3 weeks recovered to the same extent as sham mice 3 weeks after the treatment was discontinued (Figure 1B). These results indicate that ROS1 inhibition suppresses male fertility reversibly in mice.

We searched for the cause of the decreased fertility by focusing on male mice treated with lorlatinib for 3 weeks. Analysis of the testes failed to uncover any abnormalities in testicular weights (Figure S1B) or morphology of testicular cross sections (Figure S1C). Furthermore, we found no overt abnormalities in the morphology of mature spermatozoa obtained from the cauda epididymis (Figure S1D,E).

In mice, *Ros1* is expressed in the IS of the epididymis, and *Ros1* KO males exhibit IS differentiation failures while no apparent histological abnormalities were observed in the cauda epididymis.^7^, ^12^ We then performed histological analyses of the IS. The lorlatinib‐treated mice showed less thickness of the IS epithelium (Figure 1C). Furthermore, 3 weeks after discontinuation of the treatment, the thickness of the IS epithelium recovered to the same extent as in sham mice (Figure 1C). These results indicate that the administration of ROS1 inhibitors impairs IS maintenance reversibly in mature male mice.

### 3.2 In vitro fertilizing ability of spermatozoa from lorlatinib‐treated mice

**FIGURE 2:**
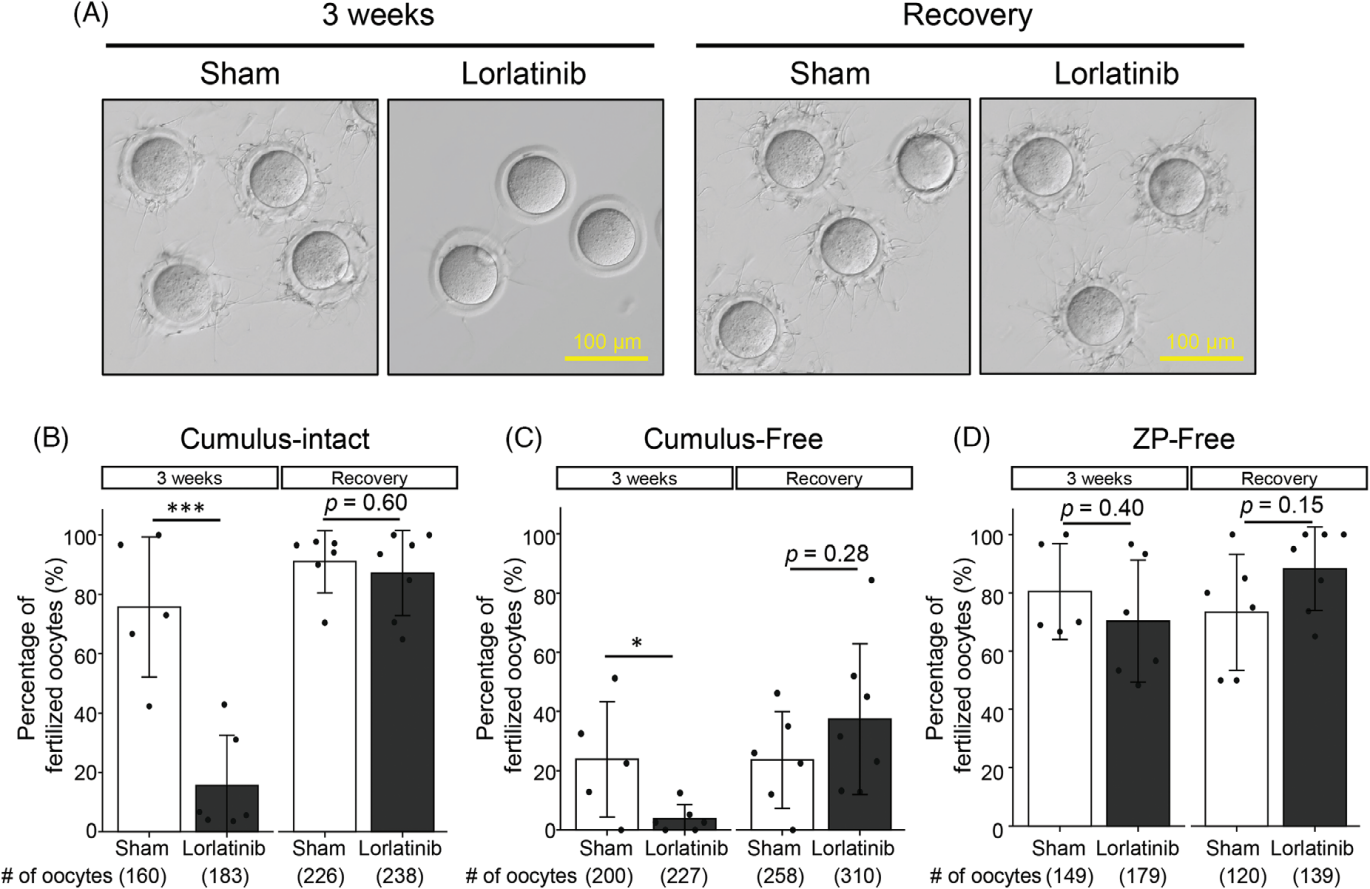
Binding ability to the zona pellucida (ZP) is impaired in spermatozoa of lorlatinib‐treated mice. (A) Sperm‐ZP binding assay was performed. (B) In vitro fertilization (IVF) with cumulus‐intact oocytes. (C) IVF with cumulus‐free oocytes. (D) IVF with ZP‐free oocytes. Data are presented as mean ± SD. Each dot indicates individual mouse. *p < 0.05, ***p < 0.001 (unpaired *t*‐test).

To further analyze the impaired male fertility of lorlatinib‐treated mice, we performe*d* IVF. It has been shown that the failure of IS epithelium differentiation results in impaired sperm maturation such as reduced spermatozoa binding to the ZP in *Ros1* KO mice.^8^ Consistently, spermatozoa of lorlatinib‐treated mice exhibit a reduced ability to bind to the ZP, while this ability is restored 3 weeks after the administration is discontinued (Figure 2A). Furthermore, consistent with impaired fertility in vivo, spermatozoa of lorlatinib‐treated mice showed reduced ability to fertilize oocytes in vitro (cumulus‐intact; Figure 2B). Removing cumulus cells could not rescue the impaired fertilization rates (cumulus‐free; Figure 2C). Alternatively, no reduction in fertilization rates was observed when both cumulus cells and ZP were removed (ZP‐free; Figure 2D). Thus, the ZP penetration ability of spermatozoa was impaired in lorlatinib‐treated mice.

### 3.3 Sperm motility in lorlatinib‐treated mice

**FIGURE 3:**
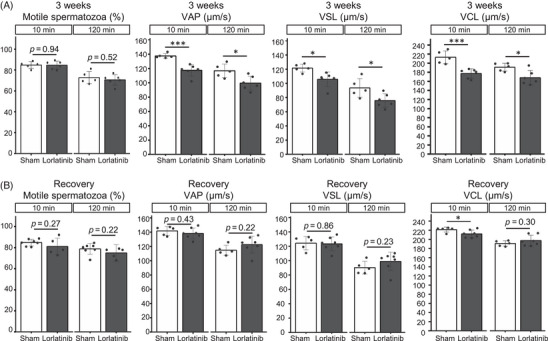
Lorlatinib‐treated mice show decreased sperm motility. Sperm motility parameters at 10 and 120 min after Toyoda, Yokoyama, Hoshi (TYH) incubation. Sperm motility was analyzed in mice treated with lorlatinib for 3 weeks (A) and after discontinuing the treatment (B). VAP, average path velocity; VCL, curvilinear velocity; VSL, straight‐line velocity. Data are presented as mean ± SD. Each dot indicates individual mouse. *p < 0.05, ***p < 0.001 (unpaired *t*‐test).

It has also been reported that *Ros1* KO mice show impaired sperm motility.^18^, ^19^ We then examined sperm motility of lorlatinib‐treated mice using a computer‐aided sperm analysis (CASA) system. Although spermatozoa were motile after 3 weeks of lorlatinib treatment, there was a significant decrease in velocity parameters such as VAP, VSL, and VCL after 10 and 120 min incubation in a capacitating medium (Figure 3A). Decreases in these velocity parameters were recovered 3 weeks after lorlatinib discontinuation (Figure 3B).

### 3.4 Immunoblot analyses of epididymal proteins in lorlatinib‐treated mice

**FIGURE 4:**
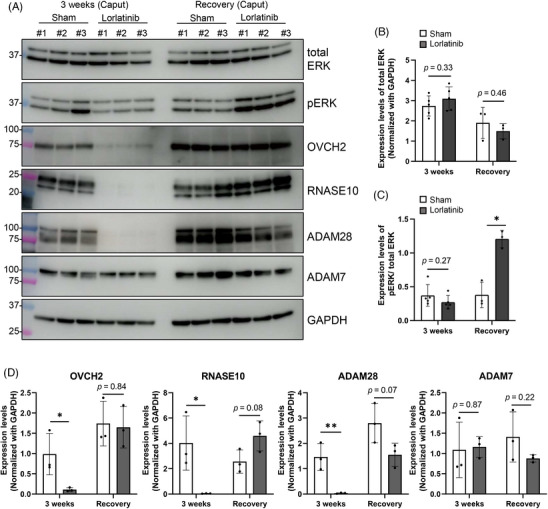
Amounts of epididymal proteins in lorlatinib‐treated mice. (A) Immunoblot detection of total extracellular signal‐regulated kinase (ERK), phospho‐ERK (pERK), OVCH2, RNASE10, ADAM28, and ADAM7 using caput epididymal lysates. Glyceraldehyde‐3‐phosphate dehydrogenase (GAPDH) was analyzed as a loading control. (B) Quantitative analyses of (A) for total ERK amounts. Signals were normalized using GAPDH. (C) Quantitative analyses of (A) for pERK amounts. Signals were normalized using total ERK. (D) Quantitative analyses of (A) for OVCH2, RNASE10, ADAM28, and ADAM7 amounts. Signals were normalized using GAPDH. Data are presented as mean ± SD. Each dot indicates individual mouse. *p < 0.05, **p < 0.01 (unpaired *t*‐test).

Extracellular signal‐regulated kinase 1/2 (ERK1/2) is a signal mediator of the mitogen‐activated protein (MAP) kinase pathway and is a downstream target of ROS1.^6^ It has been reported that phosphorylation levels of ERK1/2 are down‐regulated in *Ros1* KO epididymis.^12^ To determine whether inhibition of ROS1 has a similar effect at the molecular level we performed immunoblot analyses using caput epididymis. There was no significant difference in the amount of ERK1/2 between sham and lorlatinib‐treated mice (Figure 4A,B). Intriguingly, overt differences were not found in the amount of phosphorylated ERK1/2 as well (Figure 4A,C). It has been reported that crizotinib decreases the phosphorylation state of ERK1/2 in caput epididymis after 1 week of treatment, but that the phosphorylation state is restored after 2 weeks of treatment, suggesting that inhibition of ROS1 for a long time induces a compensatory mechanism for ERK1/2 phosphorylation.^12^ The same compensatory mechanism might be induced when lorlatinib was administered for 3 weeks. Supporting the idea of up‐regulation of the compensatory effect, the phosphorylation state of ERK1/2 was enhanced 3 weeks after discontinuation of treatment in the lorlatinib‐treated mice (Figure 4A,C).

Next, we examined the amounts of OVCH2, ADAM28, and ADAM7, which are secreted into the epididymal lumen and are related to sperm maturation.^8^, ^20^, ^21^ Consistent with *Ros1* KO mice, the amounts of OVCH2 and ADAM28, but not ADAM7, were decreased in the caput epididymis of lorlatinib‐treated mice (Figure 4A,D). Furthermore, the amount of RNASE10, another secreted protein in the epididymis, was also decreased. In addition, the amounts of OVCH2, ADAM28, and RNASE10 recovered 3 weeks after the treatment was discontinued (Figure 4A,D). Thus, lorlatinib treatment reversibly suppresses the expression of secreted proteins downstream of ROS1.

### 3.5 ADAM3 processing is abnormal in lorlatinib‐treated mice

**FIGURE 5:**
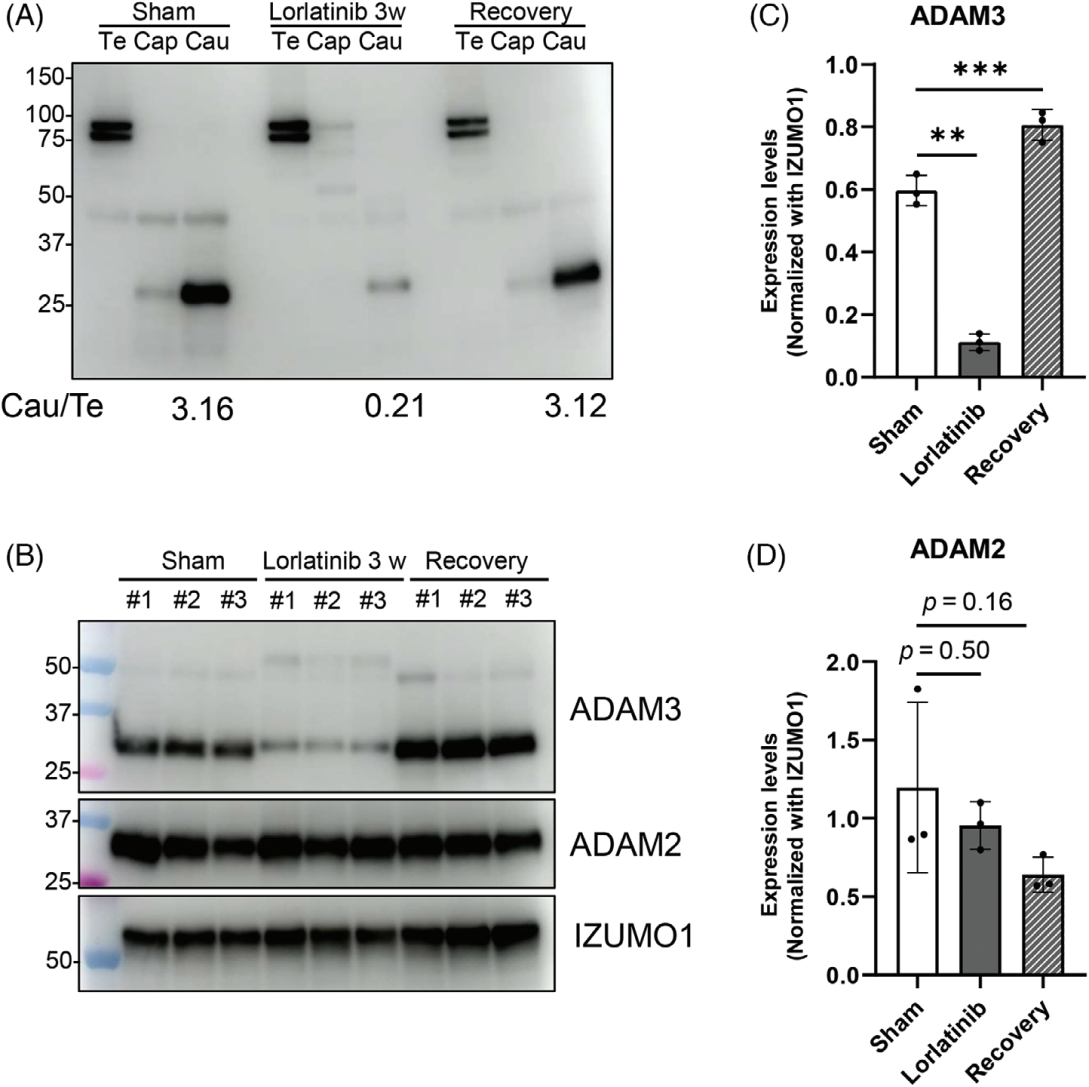
ADAM3 processing in lorlatinib‐treated mice. (A) Immunoblot detection of ADAM3 using testes, caput, and cauda epididymal lysates. Processed ADAM3 (around 30 kDa) amounts in cauda epididymis normalized by the unprocessed ADAM3 (around 90 kDa) amounts in testes were shown at the bottom. (B) Immunoblot detection of ADAM3 and ADAM2 using cauda epididymal lysates. IZUMO1 was analyzed as a loading control. (C and D) Quantitative analyses of (B) for each protein amount. Signals were normalized with IZUMO1. Data are presented as mean ± SD. Each dot indicates individual mouse. *p < 0.05, ** *p* < 0.01, ***p < 0.001 (unpaired *t*‐test).

Defective spermatozoa binding to the ZP is often associated with abnormal processing of sperm membrane protein, ADAM3.^22^ ADAM3 is expressed in round spermatids as a precursor (around 90 kDa) which is processed into mature protein (around 30 kDa) during sperm transit through the epididymis.^22^ In *Ros1* KO mice, ADAM3 processing deteriorated due to impaired secretion of OVCH2 into the epididymal lumen.^8^ To further analyze if lorlatinib‐treated mice phenocopy *Ros1* KO mice, we examined ADAM3 processing. Immunoblot analysis using testes and epididymis revealed that the amounts of processed ADAM3 around 30 kDa in the cauda epididymis decreased, indicating that ADAM3 was not correctly processed in lorlatinib‐treated mice but recovered 3 weeks after drug discontinuation (Figure 5A–C). When the amounts of other sperm membrane proteins, ADAM2 and IZUMO1, were examined,^23^, ^24^ no significant differences were found between sham and lorlatinib‐treated mice (Figure 5B,D). These results indicate that the reversible ZP binding ability of lorlatinib‐treated mice was likely due to abnormal processing of ADAM3, consistent with the findings in *Ros1* KO mice.

We then analyzed if abnormal ADAM3 processing could lead to impaired sperm motility observed in lorlatinib‐treated mice. We examined the sperm motility of *Adam3* KO mice using the CASA system and found no significant differences between control and *Adam3* KO mice (Figure S2). Thus, ZP binding defects are caused by abnormalities in ADAM3 processing, but that reduced sperm motility is caused by other mechanisms in lorlatinib‐treated mice.

## 4 DISCUSSION

Using ROS1 inhibitors, crizotinib and lorlatinib, which are used as drugs for non‐small cell lung cancer, we investigated whether ROS1 in the epididymis is an effective target for male contraceptives. Although sexually mature male mice treated with ROS1 inhibitors showed no overt abnormalities in body and testis weight, testis histology, or mating behavior, sperm function and their fertility were reversibly impaired (Figures 1B and S1A–C), indicating that ROS1 and its downstream pathway in the epididymis can be an effective target strategy for reversible contraception in males. While there is an anatomical difference between mouse and human epididymis (i.e., a thickened IS is overserved in mice but no pronounced IS in humans), the NELL2/NICOL1‐ROS1 lumicrine signaling‐related molecules are expressed in human epididymis as well,^25^ suggesting that the molecular mechanisms regulating sperm maturation may be conserved despite absence of IS differentiation in humans.

A previous study showed that crizotinib administration (100 mg/kg) for 12 days did not impair male fertility.^12^ Although there was a decrease in male fertility, our results also showed that some individuals exhibited comparable fertilization rates to sham mice after 2 weeks of lorlatinib (10 mg/kg) or crizotinib (100 mg/kg) treatment, suggesting that contraception in mice requires longer than 2 weeks of administration. Our results also showed that fertility was not completely eliminated in all mice treated with lorlatinib for 3 weeks. While five males were infertile, two individuals had reduced fertility rates of 4.2% and 25.0% (Figure 1B). Given these observations, further research is needed to optimize dosage and duration of administration to achieve more consistent and complete infertility. It is also important to explore molecules that inhibit the ROS1 pathway more efficiently.

The IS epithelium differentiation is associated with proper sperm maturation such as sperm ZP binding and sperm motility in *Ros1* KO mice.^8^, ^19^ These processes are both impaired in lorlatinib‐treated mice, which may be the cause of reduced fertility. In contrast, lorlatinib‐treated mice did not exhibit sperm flagellar angulation defects (Figure S1D,E), which has been reported in *Ros1* KO mice,^18^, ^19^ suggesting that the effect of our ROS1 inhibition is milder than that of *Ros1* KO; this milder phenotype may be due to either a hypomorphic effect of the drug inhibition or there are additional phenotypes secondary to long‐term developmental consequences in the *Ros1* KO mice.

It has been shown that the phosphorylation status of ERK1/2 is down‐regulated in *Ros1* KO mice.^12^ In contrast, significant down‐regulation of ERK1/2 phosphorylation was not found in lorlatinib‐treated mice (Figure 4A,C), while the IS epithelium maintenance was impaired (Figure 1C). One possibility to explain this discrepancy is that phosphorylated ERK1/2 may be localized abnormally in lorlatinib‐treated mice. It has been reported that after 1 week of treatment with crizotinib, the phosphorylation status of ERK1/2 decreases, but after 2 weeks of continued treatment, the phosphorylated ERK1/2 reappears.^12^ At this time, phosphorylated ERK1/2 was localized mainly to the cytoplasm instead of the nuclear localization of the control mice.^12^ Similarly, when lorlatinib is administered for 3 weeks, phosphorylated ERK1/2 may not be functional with abnormal localization, which results in impaired IS epithelium maintenance. The use of ROS1 inhibitors may help us better understand how ROS1 regulates IS epithelial maintenance via ERK1/2.

Spermatozoa from lorlatinib‐treated mice failed to bind to the ZP (Figure 2A). ADAM3, a sperm membrane protein, is important for ZP binding,^16^ and it is known that ADAM3 processing is abnormal in *Ros1* KO mice.^8^ It is also known that *Adam3* KO spermatozoa cannot bind to the ZP but can fertilize oocytes in IVF if cumulus cell layers are present.^22^ In contrast, spermatozoa from lorlatinib‐treated mice failed to fertilize oocytes even in the presence of cumulus cell layers (Figure 2A). This may be due to reduced sperm motility combined with impaired ZP binding in lorlatinib‐treated mice (Figure 2A). It has been shown that OVCH2 plays roles in ADAM3 processing,^8^ but it is still unclear how sperm motility is regulated in the IS.

In summary, we revealed that the inhibition of ROS1 by lorlatinib leads to abnormal sperm maturation and reduced male fertility in mature mice, which recovers after the discontinuation of the drug administration. No obvious abnormalities other than male infertility were observed in *Ros1* KO mice,^7^ but it is unclear whether specific ROS1 inhibition has adverse effects in humans. In clinical practice, lorlatinib (targets: ALK/ROS1) is associated with primarily mild to moderate side effects, including hypercholesterolemia (82.4%), hypertriglyceridemia (60.7%), edema (51.2%), peripheral neuropathy (43.7%), and central nervous system effects (39.7%),^26^ but edema has also been reported with alectinib (targets: ALK/RTK),^27^ suggesting that specific inhibition of ROS1 but not ALK may reduce edema. Further, it has been shown that hypercholesterolemia, hypertriglyceridemia, and cognitive effects were more frequently observed with lorlatinib than with crizotinib (targets: ALK/ROS1),^28^ suggesting that these side effects may be caused by off‐target inhibition of lorlatinib. We showed that the level of OVCH2 decreased in lorlatinib‐treated mice (Figure 4A). A search for molecules that specifically inhibit ROS1 or its downstream molecules, such as OVCH2, may pave the way for the development of safer and more effective male contraceptives.

## AUTHOR CONTRIBUTIONS

All the authors designed the study. Yuki Oyama, Kentaro Shimada, Haruhiko Miyata, Rie Iida‐Norita, Chihiro Emori, Maki Kamoshita, and Seiya Oura performed experiments. All the authors analyzed the data. Yuki Oyama, Haruhiko Miyata, Martin M. Matzuk, and Masahito Ikawa wrote the manuscript draft. All the authors edited and approved the final manuscript.

## CONFLICT OF INTEREST STATEMENT

The authors declare no conflicts of interest.

## Supporting information



Supporting Information

## Data Availability

The data that support the findings of this study are available from the corresponding author upon reasonable request.

## References

[1] Bearak J , Popinchalk A , Ganatra B , et al. Unintended pregnancy and abortion by income, region, and the legal status of abortion: estimates from a comprehensive model for 1990–2019. Lancet Glob Health. 2020;8(9):e1152‐e1161. doi:10.1016/S2214-109X(20)30315-6 32710833

[2] Matzuk MM , McKeown MR , Filippakopoulos P , et al. Small‐molecule inhibition of BRDT for male contraception. Cell. 2012;150(4):673‐684. doi:10.1016/j.cell.2012.06.045 22901802 PMC3420011

[3] Ku AF , Sharma KL , Ta HM , et al. Reversible male contraception by targ1eted inhibition of serine/threonine kinase 33. Science. 2024;384(6698):885‐890. doi:10.1126/science.adl2688. Epub 2024 May 23. 38781365 PMC11842024

[4] Balbach M , Rossetti T , Ferreira J , et al. On‐demand male contraception via acute inhibition of soluble adenylyl cyclase. Nat Commun. 2023;14(1):637. doi:10.1038/s41467-023-36119-6 36788210 PMC9929232

[5] Miyata H , Satouh Y , Mashiko D , et al. Sperm calcineurin inhibition prevents mouse fertility with implications for male contraceptive. Science. 2015;350(6259):442‐445. doi:10.1126/science.aad0836 26429887

[6] Drilon A , Jenkins C , Iyer S , et al. ROS1‐dependent cancers‐biology, diagnostics and therapeutics. Nat Rev Clin Oncol. 2021;18(1):35‐55. doi:10.1038/s41571-020-0408-9 32760015 PMC8830365

[7] Sonnenberg‐Riethmacher E , Walter B , Riethmacher D , et al. The c‐ros tyrosine kinase receptor controls regionalization and differentiation of epithelial cells in the epididymis. Genes Dev. 1996;10(10):1184‐1193. doi:10.1101/gad.10.10.1184 8675006

[8] Kiyozumi D , Noda T , Yamaguchi R , et al. NELL2‐mediated lumicrine signaling through OVCH2 is required for male fertility. Science. 2020;368(6495):1132‐1135. doi:10.1126/science.aay5134 32499443 PMC7396227

[9] Kiyozumi D , Shimada K , Chalick M , et al. A small secreted protein NICOL regulates lumicrine‐mediated sperm maturation and male fertility. Nat Commun. 2023;14(1):2354. doi:10.1038/s41467-023-37984-x 37095084 PMC10125973

[10] Fawcett DW , Hoffer AP . Failure of exogenous androgen to prevent regression of the initial segments of the rat epididymis after efferent duct ligation or orchidectomy. Biol Reprod. 1979;20(2):162‐181. doi:10.1095/biolreprod20.2.162 454730

[11] Kim B , Roy J , Shum WW , et al. Role of testicular luminal factors on basal cell elongation and proliferation in the mouse epididymis. Biol Reprod. 2015;92(1):9. doi:10.1095/biolreprod.114.123943 25411392 PMC4434934

[12] Jun HJ , Roy J , Smith TB , et al. ROS1 signaling regulates epithelial differentiation in the epididymis. Endocrinology. 2014;155(9):3661‐3673. doi:10.1210/en.2014-1341 24971615 PMC4138574

[13] Toyoda Y , Yokoyama M , Hosi T . Studies on the fertilization of mouse eggs in vitro. Jpn J Anim Reprod. 1971;16:152‐157.

[14] Miyata H , Oura S , Morohoshi A , et al. SPATA33 localizes calcineurin to the mitochondria and regulates sperm motility in mice. Proc Natl Acad Sci USA. 2021;118(35):e2106673118. doi:10.1073/pnas.2106673118 34446558 PMC8536318

[15] Oyama Y , Miyata H , Shimada K , et al. TULP2 deletion mice exhibit abnormal outer dense fiber structure and male infertility. Reprod Med Biol. 2022;21(1):e12467. doi:10.1002/rmb2.12467 35619658 PMC9126596

[16] Yamaguchi R , Yamagata K , Ikawa M , et al. Aberrant distribution of ADAM3 in sperm from both angiotensin‐converting enzyme (Ace)‐ and calmegin (Clgn)‐deficient mice. Biol Reprod. 2006;75:760‐766. doi:10.1095/biolreprod.106.052977 16870943

[17] Morohoshi A , Miyata H , Oyama Y , et al. FAM71F1 binds to RAB2A and RAB2B and is essential for acrosome formation and male fertility in mice. Development. 2021;148(21):dev199644. doi:10.1242/dev.199644 34714330 PMC8602946

[18] Yeung CH , Sonnenberg‐Riethmacher E , Cooper TG . Infertile spermatozoa of c‐ros tyrosine kinase receptor knockout mice show flagellar angulation and maturational defects in cell volume regulatory mechanisms. Biol Reprod. 1999;61(4):1062‐1069. doi:10.1095/biolreprod61.4.1062 10491645

[19] Yeung CH , Wagenfeld A , Nieschlag E , et al. The cause of infertility of male c‐ros tyrosine kinase receptor knockout mice. Biol Reprod. 2000;63(2):612‐618. doi:10.1095/biolreprod63.2.612 10906072

[20] Kent K , Nozawa K , Sutton C , et al. CUB domains are not required for OVCH2 function in sperm maturation in the mouse epididymis. Andrology. 2024;12(3):682‐697. doi:10.1111/andr.13508 37551853 PMC10850435

[21] Choi H , Han C , Jin S , et al. Reduced fertility and altered epididymal and sperm integrity in mice lacking ADAM7. Biol Reprod. 2015;93(3):70. doi:10.1095/biolreprod.115.130252 26246218

[22] Tokuhiro K , Ikawa M , Benham AM , et al. Protein disulfide isomerase homolog PDILT is required for quality control of sperm membrane protein ADAM3 and male fertility. Proc Natl Acad Sci U S A. 2012;109(10):3850‐3855. doi:10.1073/pnas.1117963109 22357757 PMC3309714

[23] Cho C , Bunch DO , Faure JE . Fertilization defects in sperm from mice lacking fertilin beta. Science. 1998;281(5384):1857‐1859. doi:10.1126/science.281.5384.1857 9743500

[24] Inoue N , Ikawa M , Isotani A , et al. The immunoglobulin superfamily protein Izumo is required for sperm to fuse with eggs. Nature. 2005;434(7030):234‐238. doi:10.1038/nature03362 15759005

[25] Kiyozumi D . Expression of NELL2/NICOL‐ROS1 lumicrine signaling‐related molecules in the human male reproductive tract. Reprod Biol Endocrinol. 2024;22(1):3. doi:10.1186/s12958-023-01175-6 38169386 PMC10759339

[26] Bauer TM , Felip E , Solomon BJ , et al. Clinical management of adverse events associated with lorlatinib. Oncologist. 2019;24(8):1103‐1110. doi:10.1634/theoncologist.2018-0380 30890623 PMC6693708

[27] Camidge DR , Dziadziuszko R , Peters S , et al. Updated efficacy and safety data and impact of the EML4‐ALK fusion variant on the efficacy of alectinib in untreated ALK‐positive advanced non‐small cell lung cancer in the global phase III ALEX study. J Thorac On col. 2019;14(7):1233‐1243. doi:10.1016/j.jtho.2019.03.007 30902613

[28] Shaw AT , Bauer TM , de Marinis F , et al. First‐line lorlatinib or crizotinib in advanced ALK‐positive lung cancer. N Engl J Med. 2020;383(21):2018‐2029. doi:10.1056/NEJMoa2027187 33207094

