# Supplementary material for: Inhibition of ROS1 activity with lorlatinib reversibly suppresses fertility in male mice
Source: Andrology. 2024 Nov 20;13(7):1891–900. doi: 10.1111/andr.13808 (PMC12476209; doi:10.1111/andr.13808)
Supplement: Supplementary file 1 — Supporting Information [file ANDR-13-1891-s001.pdf]

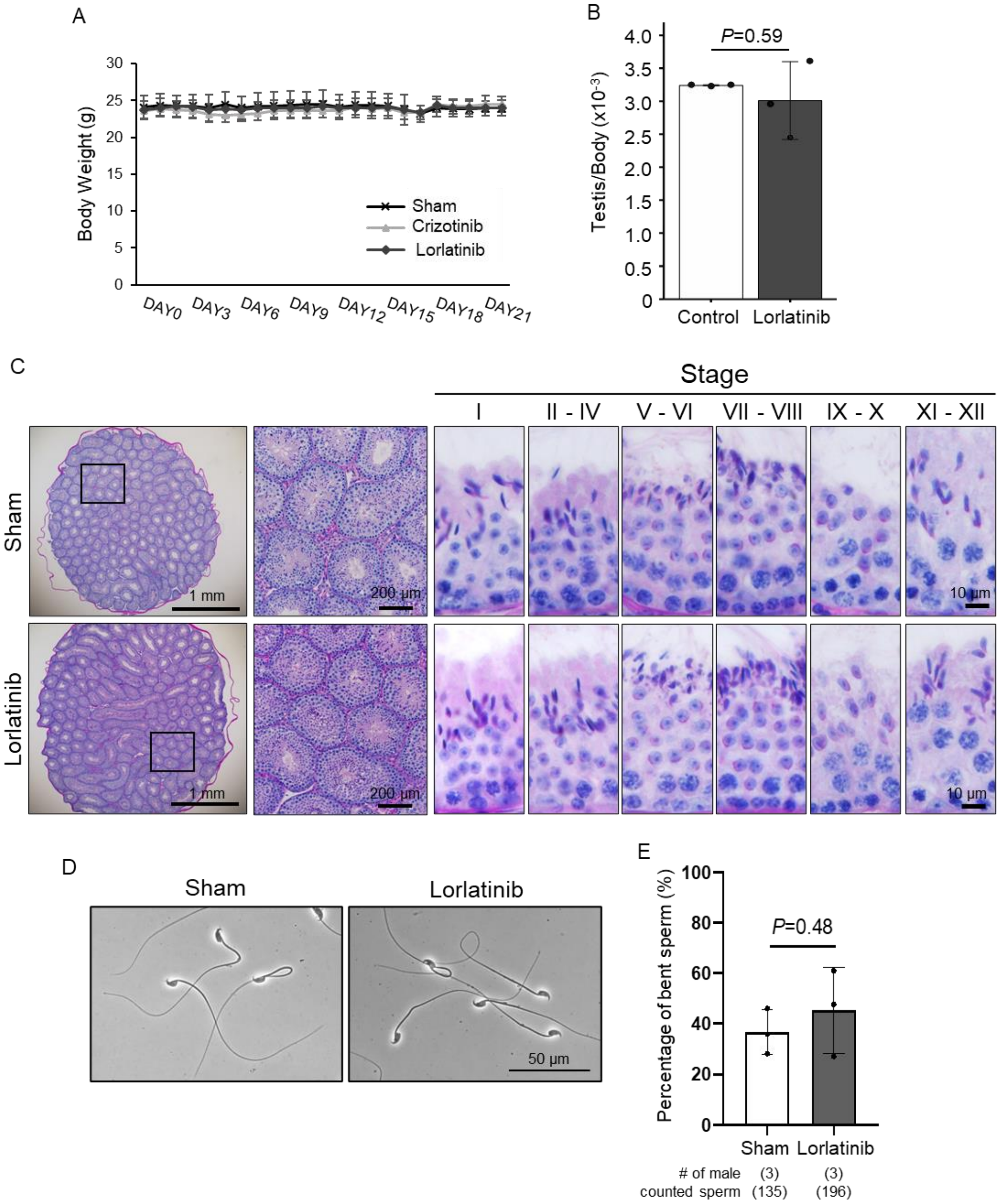

Figure S1. Testis sections and sperm morphology of drug-treated mice. (A) Body weight during the drug treatment. Nine male mice were used for the analysis. (B) Testicular weight per body weight. Data are presented as mean ± SD. Each dot indicates individual mouse. There was no significant difference (unpaired *t*-test). (C) PAS-H staining of testis sections. (D) Sperm morphology obtained from the cauda epididymis. (E) Percentage of spermatozoa with bent tails. Data are presented as mean ± SD. Each dot indicates individual mouse. There was no significant difference (unpaired *t*-test).

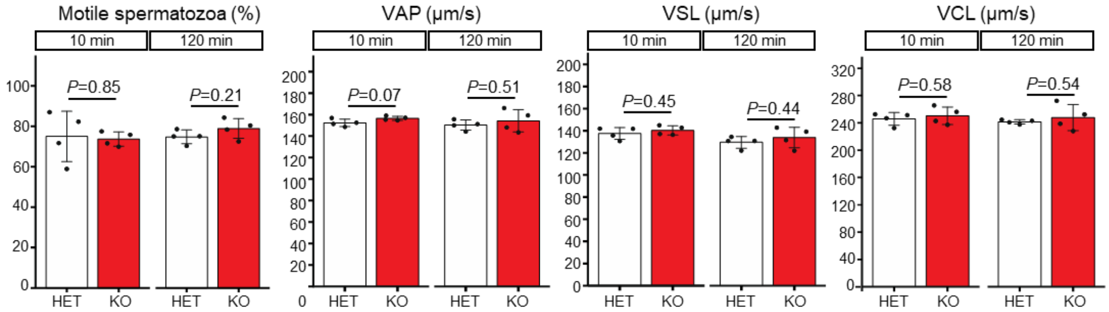

Figure S2. Sperm motility of *Adam3* KO mice. Sperm motility was analyzed at 10 min and 120 min after TYH incubation in *Adam3* KO mice. VAP, average path velocity; VCL, curvilinear velocity; VSL, straight-line velocity. Data are presented as mean ± SD. Each dot indicates individual mouse. There was no significant difference (unpaired *t*-test).

Table S1. Antibodies used in this study.

Antibodies	Source	Identifier	Dilution
ADAM2	Sigma-Aldrich	MAB19292	1:1000
ADAM3	Santa Cruz	sc-365288	1:1000
ADAM28	proteintech	22234-1-AP	1:500
ADAM7	Sigma-Aldrich	HPA008879	1:1000
GAPDH	Cell Signaling Technology	#2118	1:2000
IZUMO1	(Ikawa et al., 2011)	#125	1:1000
OVCH2	(Kiyozumi et al., 2020)		1:500
p44/42 MAPK (Erk1/2)	Cell Signaling Technology	#4695	1:1000
Phospho-p44/42 MAPK (Erk1/2) (Thr202/Tyr204)	Cell Signaling Technology	#4370	1:1000
RNASE10	LifeSpan BioSciences	LS-C296261	1:400
Rabbit IgG (HRP conjugated)	Jackson Immuno Research	111-036-045	1:5000
Rat IgG (HRP conjugated)	Jackson Immuno Research	112-035-167	1:5000
Mouse IgG (HRP conjugated)	Jackson Immuno Research	115-036-062	1:5000

Supporting Information References

Ikawa H, Tokuhiro K, Yamaguchi R, et al. Calsperin is a testis-specific chaperone required for sperm fertility. *J Biol Chem*. 2011;**286**(7):5639-46. doi: 10.1074/jbc.M110.140152.

Kiyozumi D, Noda T, Yamaguchi R, et al. NELL2-mediated lumicrine signaling through OVCH2 is required for male fertility. *Science*. 2020;**368**(6495):1132-1135. doi:10.1126/science.aay5134.
